# Identification of hub genes associated with follicle development in multiple births sheep by WGCNA

**DOI:** 10.3389/fvets.2022.1057282

**Published:** 2022-12-19

**Authors:** Jinglei Wang, Hanying Chen, Xiancun Zeng

**Affiliations:** ^1^College of Animal Science and Technology, Shihezi University, Shihezi, China; ^2^School of Pharmacy, Shihezi University, Shihezi, China

**Keywords:** fecundity, ovaries, mRNA, Qira black sheep, WGCNA

## Abstract

Sheep exhibit a distinct estrous cycle that includes four different phases: proestrus, estrus, late estrus, and luteal phase. As the estrous cycle repeats, follicular development regularly alternates. We thus investigated ovarian transcriptome data from each of the four phases using weighted gene co-expression network analysis (WGCNA) to identify modules, pathways, and genes essential to follicle growth and development. We clustered mRNA and long non-coding RNA (lncRNA) into different modules by WGCNA, and calculated correlation coefficients between genes and Stages of the estrous cycle. Co-expression of the black module (cor = 0.81, *P*<0.001) and the yellow module (cor = 0.61, *P*<0.04) was found to be critical for follicle growth and development. A total of 2066 genes comprising the black and yellow modules was used for functional enrichment. The results reveal that these genes are mainly enriched in Cell cycle, PI3K-Akt signaling pathway, Oocyte meiosis, Apoptosis, and other important signaling pathways. We also identified seven hub genes (*BUB1B, MAD2L1, ASPM, HSD3B1, WDHD1, CENPA*, and *MXI1*) that may play a role in follicle development. Our study may provide several important new markers allowing in depth exploration of the genetic basis for multiparous reproduction in sheep.

## Introduction

The Cele Black sheep is an exCelent lambskin sheep in Xinjiang, China. It is characterized by a high reproduction rate, year-round estrus, and an average lambing rate of 215.6% (1). It is an ideal model for studying and identifying genes related to multiparous traits. Follicle development is a crucial factor affecting multiparous traits in sheep, and improving the multiparous capacity in sheep will effectively enhance the efficiency of animal production and reproduction. As the basic functional unit of the ovary (2), the follicle consists of oocytes, which are surrounded by theca and granulosa cells (GC), and biological processes such as development, maturation, and atresia alternately generated during the estrous cycle (3). The interaction of oocyte and granulosa cells in early follicular stages has been demonstrated, and the specificity of oocyte-secreted steroid hormones and intraovarian regulators jointly determine follicular development. The balance of ovarian granulosa cell proliferation and apoptosis also plays an essential role in follicle dominance selection (4, 5). Therefore, identifying genes related to follicular development is crucial for studying multiparous traits in sheep. In the past, it has been reported that mutations in the Booroola fecundity (*Fec*^*B*^*)* gene associated with sheep reproduction lead to the inactivation of the BMPR1B protein and impair BMP signaling, thereby regulating ovarian granulosa cell differentiation, increasing ovulation rate and litter size (6, 7). Intensive research has led to the identification of several genes related to fecundity, such as KLF transcription factor 5(*KLF5*) (8), myosin heavy chain 15 (*MYH15*) (9), growth differentiation factor 9 (*GDF9*) (10), and bone morphogenetic protein-15 (*BMP15*) genes (11).

Long non-coding RNAs (LncRNAs) regulate gene expression through multiple mechanisms and are involved in follicle maturation. For example, LncRNA affects promoter activity and regulates ovarian granulosa cell proliferation and follicle maturation (12, 13). LncRNA can also act as a competitive endogenous RNA for miRNA, interacting with miRNA to regulate gene expression and affect ovarian granulosa cell proliferation (14). In recent years, lncRNA expression profiles have been obtained in the ovaries of mammalian goats (Anhui White Goat) (15) and sheep (Dorset sheep, Small-tailed Han Sheep) (9, 16) and indicated that they are reproductively important lncRNAs.

The rapid development of high-throughput sequencing technologies has improved our knowledge and insights into molecular biology. The ease of use and comprehensiveness of weighted gene co-expression network analysis (WGCNA) has been applied by researchers in fields including medicine, microbiology, and zoology. The WGCNA algorithm uses the inter-molecular relationship to simplify complex data into different classes of modules (17, 18). Molecular expression patterns of the genes of the same module may help identify the genes that play the same role or participate in the same signaling pathway and finally help discover the biological significance of these genes (19). WGCNA has made tremendous progress in identifying genes implicated in reproduction in mammals. It has been reported that *VAV1, RUNX3, ZC3H12D, MYCL, IRF5, WEE2*, and miR-3940-5P play critical roles in oocyte development and ovarian granulosa cell proliferation (20–22). In recent years, studies on the application of WGCNA on sheep fertility data have shown that *AKT3* and lncRNA genes, including *NR0B1, XLOC_041882*, and *MYH15*, are essential for follicle growth, oocyte maturation, and ovulation, being closely related genes in sheep reproduction (23, 24).

Previous studies by the project team have demonstrated that crucial reproductive genes, including *FecB, GDF9*, and *BMP15*, are not significantly differentially expressed in Cele black sheep. Therefore, it is speculated that other genes act as potential regulators to alter the expression patterns of reproduction-related genes, thereby affecting the multiparity of Cele black sheep (25). Follicular development is regulated by an extensive network of cytokines, and the associated gene expression may change depending on the stage of the estrous cycle. Therefore, we applied the WGCNA algorithm to the analysis of the ovarian transcriptome data of Cele black sheep to identify co-expressed module genes associated with follicular development at various stages of the estrous cycle of high-yielding sheep, and whose function may affect multiparity traits. This study provides a valuable resource for better understanding the molecular mechanisms of genes regulating sheep reproduction at different physiological stages.

## Materials and methods

### Ethical statement

Cele black sheep were purchased from a sheep farm in Cele County, Hotan Region, Xinjiang Uygur Autonomous Region, China. All experiments were conducted under the relevant guidelines and regulations established by the Ministry of Agriculture of the People's Republic of China. The Animal Experiment Ethics Committee of the First Affiliated Hospital of the Shihezi University School of Medicine approved all experimental procedures (A2016-085).

### Laboratory animal samples and collection

Twelve Cele black ewes of similar age, between 3 and 4 years, were selected based on breeding records, age, and body size. The 12 ewes were synchronized to estrus by injecting human synthetic progesterone, and the ewes were tested daily for artificial and ram estrus. Then, after the third estrus, the two ovaries of the ewes were collected in groups: 7 days after the end of estrus, 14 days after the end of estrus, day 1 of estrus, and days 2–3 of estrus. The estrous cycle was divided into the luteal stage, pre-estrus, estrus, and late estrus, and three replicate samples were selected from each group and named sequentially (QP1, QP2, QP3, QE1, QE2, QE3, QD1, QD2, QD3, QM1, QM2, and QM3). Tubes containing test samples were immediately frozen in liquid nitrogen and finally stored at −80°C for subsequent experiments.

### Total RNA library construction and sequencing

Total tissue RNA was extracted using TRIzol reagent (Invitrogen™, Carlsbad, CA, USA) according to the manufacturer's manual, and the extracted RNA was tested for quality and integrity (Agilent Technologies, CA, USA). Next, rRNA was removed using the Epicenter-Ribo-zero rRNA Removal Kit (Epicentre, USA), and rRNA-free residues were removed by ethanol precipitation. Then, sequencing libraries were generated using NEBNext Ultra Directed RNA Library Preparation Kit for Illumina (NEB, USA). The library was sequenced at Novogene (Beijing, China) using the Illumina Hiseq 4000 base platform, and 150 bp paired-end reads were generated.

### Raw data quality control and transcript assembly

Poor-quality bases and adaptor sequences were removed from the raw data using FastQC software (v0.11.9) to obtain clean reads. The reference genome index was constructed using bowtie2 (v2.2.8), and paired-end clean reads were aligned to the reference genome using HISAT2 (v2.2.1), followed by the reference sheep genome (http://ftp.ensembl.org/pub/release-103/fasta/ovis_aries1) comparison analysis. StringTie (http://ccb.jhu.edu/software/stringtie/index.shtml?t=manual) assembled mapped reads for each sample.

### Data analysis

#### lncRNA screening

The screening process involved the following steps: (1) Filter out a large number of low-expression and low-confidence single-exon transcripts in the transcript splicing results, and select transcripts with exon number ≥2; (2) Select transcripts with a transcript length > 200 bp; (3) Use Cuffcompare software to screen out transcripts that overlap with the exon region annotated in the database and put the transcripts in the database that overlap with the exon region of this spliced transcript, fragments per kilobase per million reads (FPKM) of lncRNAs ≥ 0.5 were included in the subsequent analysis as database annotation lncRNAs; (4) Finally, the analysis was performed using the coding potential calculator (score < 0) (26), coding-noncoding index (score < 0) (27), and Pfam (*E*-value < 0.001) (28) software. Transcripts that pass all these stages are considered lncRNAs. Expression levels of lncRNAs are reflected in FPKM.

#### Differential expression analysis

The Ballgown suite includes functions for interactive exploration of the transcriptome assembly, visualization of transcript structures and feature-specific abundances for each locus, and post-hoc annotation of assembled features to annotated features. Transcripts with an *P*-adjust < 0.05 were assigned as differentially expressed.

### Weighted gene co-expression network analysis

#### Weighted gene co-expression network construction

We constructed a co-expression network using the WGCNA algorithm under the R package and RStudio (v 4.1.0) environment (https://horvath.genetics.ucla.edu/html/CoexpressionNetwork/Rpackages/WGCNA/). First, the genes with the highest average FPKM values were selected from the analyzed samples after using the Pearson correlation matrix and the average linkage method, using the power function A_mn=|C_mn|^∧^β (C_mn = Pearson correlation between Gene_m and Gene_n; A_mn = adjacency between gene m and gene n). β is a soft threshold parameter that can emphasize strong correlations between genes and penalize weak correlations. After selecting the power of the βvalue, the adjacency was transformed into a topological overlap matrix (TOM), which can measure the network connectivity of a gene, defined as the sum of its adjacencies with all other genes, which is used for network generation, and the corresponding dissimilarity (1-TOM) is calculated.

#### Module-feature correlation analysis and identification of modules of interest

Genes with similar expression profiles were classified into gene modules, and average linkage hierarchical clustering was performed according to the TOM-based dissimilarity measure, with the sensitivity set to 3. Based on the dissimilarity of the module eigengenes (ME), we selected a cutting line for the module dendrogram, merging a few modules. We performed a correlation analysis between each module and each stage of the estrous cycle to unearth the relevant modules highly related to follicle development. Subsequently, intra-module analysis was performed using gene significance (GS) and module membership (MM). GS signifies the relationship between gene expression levels and the stages of the estrous cycle of sheep, whereas MM represents the association between the gene expression profile of a given module and ME. Modules containing genes with significant correlations between GS and MM were considered significant.

### Functional enrichment analysis

LncRNA target genes and differentially expressed mRNAs (DEmRNAs) were subjected to GO and KEGG enrichment analysis using the KOBAS online database (http://kobas.cbi.pku.edu.cn/). Gene set enrichment analysis (GSEA) is a method to identify functional pathways. Using R package functions “ClusterProfiler”, “GSEABase”, and RStudio (v4.1.0) for GSEA, genes were ranked briefly based on the absolute value of logFC between each genome of sheep in the QD, QE, QM, and QP groups, and enrichment scores were calculated. Next, we fed the sorted list of genes into the GSEA algorithm to correlate gene expression with functional enrichment. The criteria for statistical significance were at *p* < 0.05 and FDR < 0.25.

### Construction of PPI network construction and identification of hub genes

We searched through the Search Tool for Interacting Genes/Proteins (STRING) database at (https://string-db.org/) for the construction of the protein-protein interaction (PPI) network. The network graph was visualized and analyzed using the MCODE plugin in Cytoscape (v3.7.1) for highly connected hub proteins throughout the network. Ultimately, overlapping genes among PPI hub proteins, intra-module genes, and differential genes were identified as key candidate genes for regulating follicle development.

### Transcription factor analysis transcription factor analysis

AnimalTFDB (http://bioinfo.life.hust.edu.cn/AnimalTFDB/#!) database was used for transcription factor prediction. In addition, control options were set by Blast *E*-value (1.0E-5) and selection of Hummsacn *E*-value (1.0E-5).

## Results

### Assembly of RNA-seq data

A total of 1,367,886,468 raw reads were obtained by sequencing all samples QD (QD1, QD2, QD3), QE (QE1, QE2, QE3), QM (QM1, QM2, QM3), QP (QP1, QP2, QP3) 12 libraries. After removing redundant and low-quality reads, 1,330,939,226 clean single-ended reads were obtained. The mapping rate ranged from 97.15 to 97.85%, the Q30 ratio was above 93.23%, and the GC content was above 47.17% (Supplementary Table S1).

### Identification of differential lncRNA, mRNA

**Figure 1:**
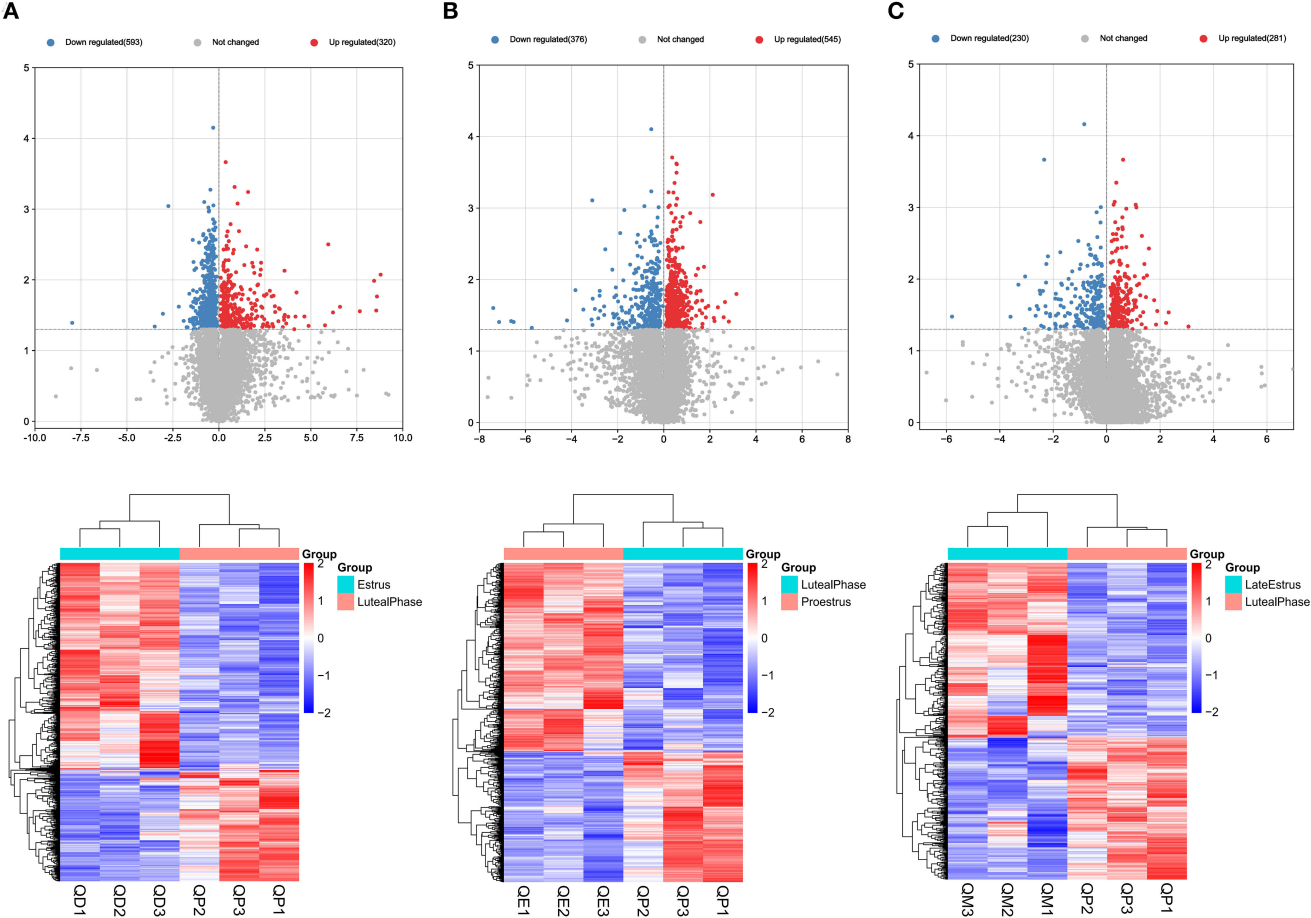
Graphical representation of DEmRNAs at each stage of the estrous cycle in sheep. The Volcano map and Hierarchical clustering analysis of DEmRNAs in the QD-QP **(A)**, QE-QP **(B)**, and QM-QP **(C)**.

Gene expression levels were quantified using FPKM. Volcano plots were used to illustrate all DEmRNAs and DElncRNAs in the genome between QE and QM, QD and QE, QD and QM, QD and QP, QE and QP, and QM and QP. Based on gene differential expression levels, there were significant differences between QD and QP, QE and QP, and QM and QP, with 913, 921, and 511 detected DEmRNAs, respectively (Figures 1A–C). A total of 298, 248, and 245 DEmRNAs were identified in the QD-QE, QD-QM, and QE-QM groups, respectively (Supplementary Figure S1). In the six comparison groups, we obtained a total of 2245 DEmRNAs by screening criteria (**Figure 3A**, Supplementary Table S2).

**Figure 2:**
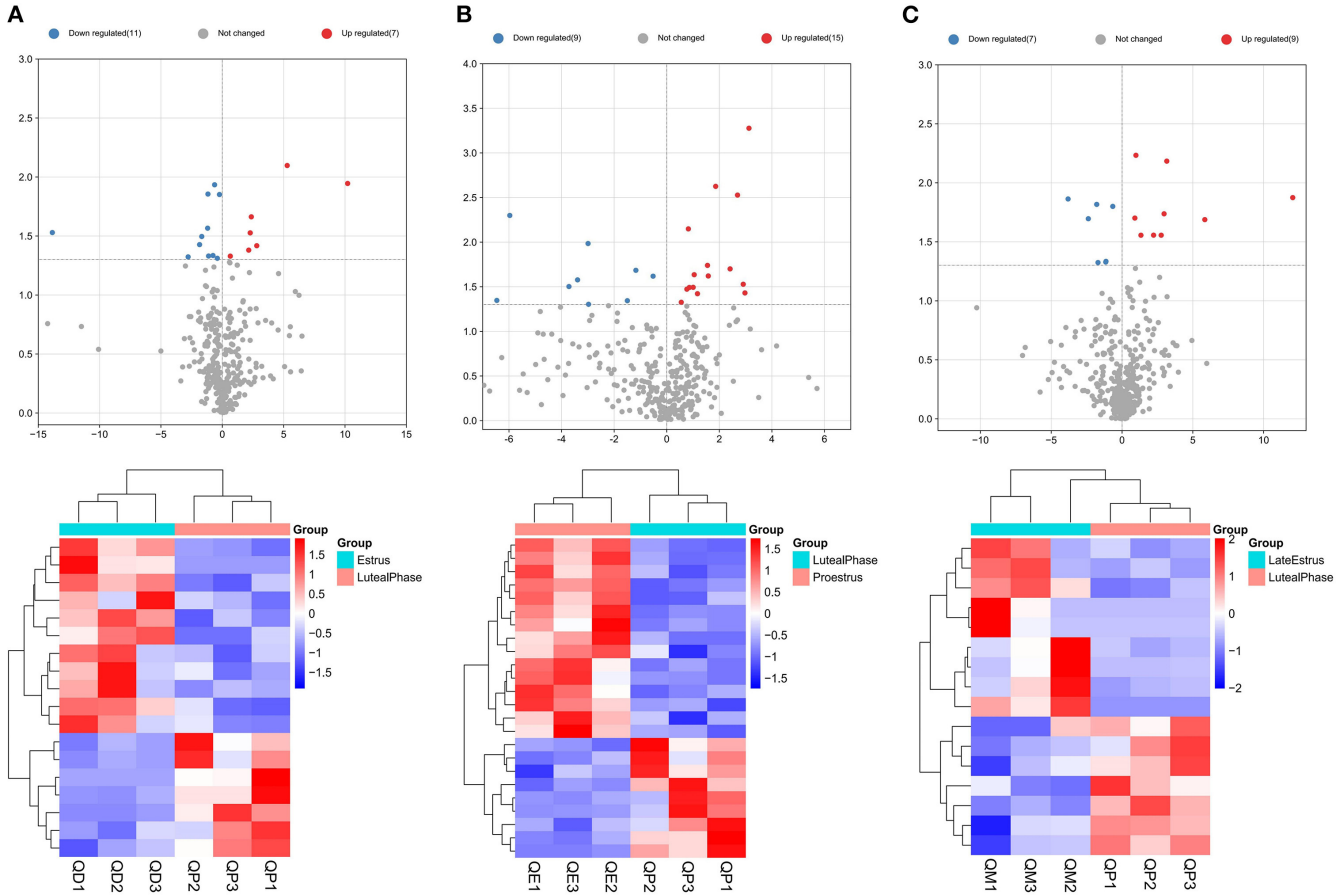
Graphical representation of DELncRNAs at each stage of the estrous cycle in sheep. The Volcano map and Hierarchical clustering analysis of DELncRNAs in the QD-QP **(A)**, QE-QP **(B)**, and QM-QP **(C)**.

**Figure 3:**
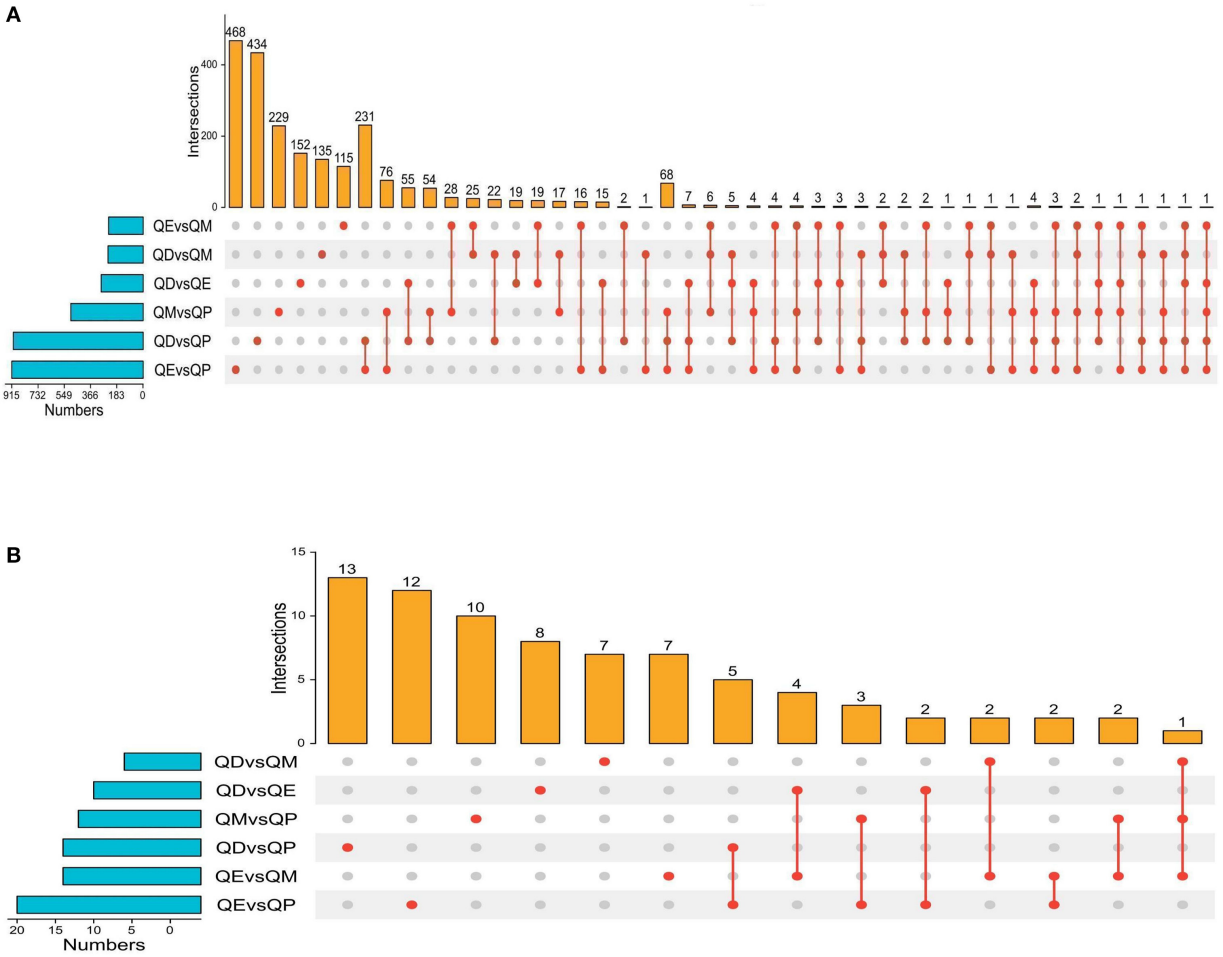
Upset plot diagram of the gene comparison result. **(A)** Upset plot diagram was employed to intersect the six gene sets and obtained 2245 common DEmRNAs. **(B)** Upset plot diagram of all DELncRNAs numbers among the six gene sets.

Eighteen, 24, and 16 DElncRNAs were detected in QD-QP, QE-QP, and QM-QP comparisons, respectively (Figures 2A–C). Fourteen, ten, and eighteen DElncRNAs were found between the QD-QE, QD-QM, and QE-QM groups, respectively (Supplementary Figure S2). In the six comparison groups, we obtained a total of 78 DELncRNAs by screening criteria (Figure 3B, Supplementary Table S3).

### Functional enrichment analysis

**Figure 4:**
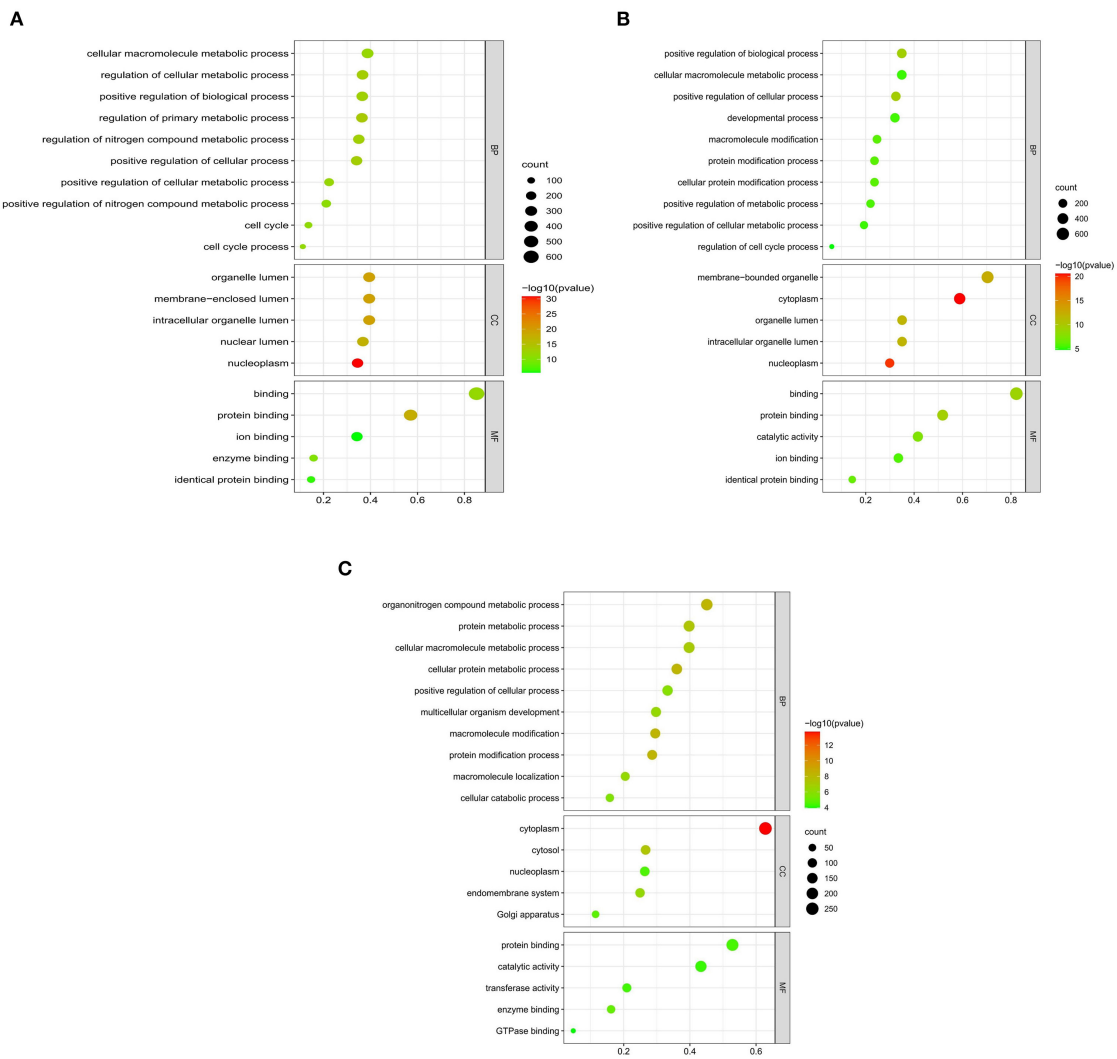
GO enrichment analysis of DElncRNAs and DEmRNAs. **(A)** QD-QP. **(B)** QE-QP. **(C)** QM-QP.

We searched coding genes 100 k upstream and downstream of lncRNA and took the intersection with the genes that had significant co-expression with these lncRNAs. As a result, 56 DElncRNAs had to target regulatory relationships with 145 genes (Supplementary Table S4). In this context, we used the KOBAS online database for GO and KEGG pathway annotation analysis. GO enrichment analysis showed that in the QD-QP groups, genes were mostly enriched in the regulation of the primary metabolic process, regulation of the cellular metabolic process, positive regulation of cellular process, cell cycle and positive regulation of biological process (Figure 4A). In the QE-QP groups, these DEmRNAs were significantly enriched in the positive regulation of cellular process, positive regulation of biological process, cellular protein modification process, regulation of cell cycle process (Figure 4B). In the QM-QP groups, genes were significantly enriched in processes such as cellular macromolecule metabolic process, cellular catabolic process, positive regulation of cellular process and multicellular organism development (Figure 4C). In addition, differential genes were significantly enriched in QD-QM, QE-QM, and QD-QE for cell cycle process, cellular macromolecule metabolic process, cellular macromolecule biosynthetic process, cellular biosynthetic process, cell migration, and cell motility (Supplementary Figure S3).

**Figure 5:**
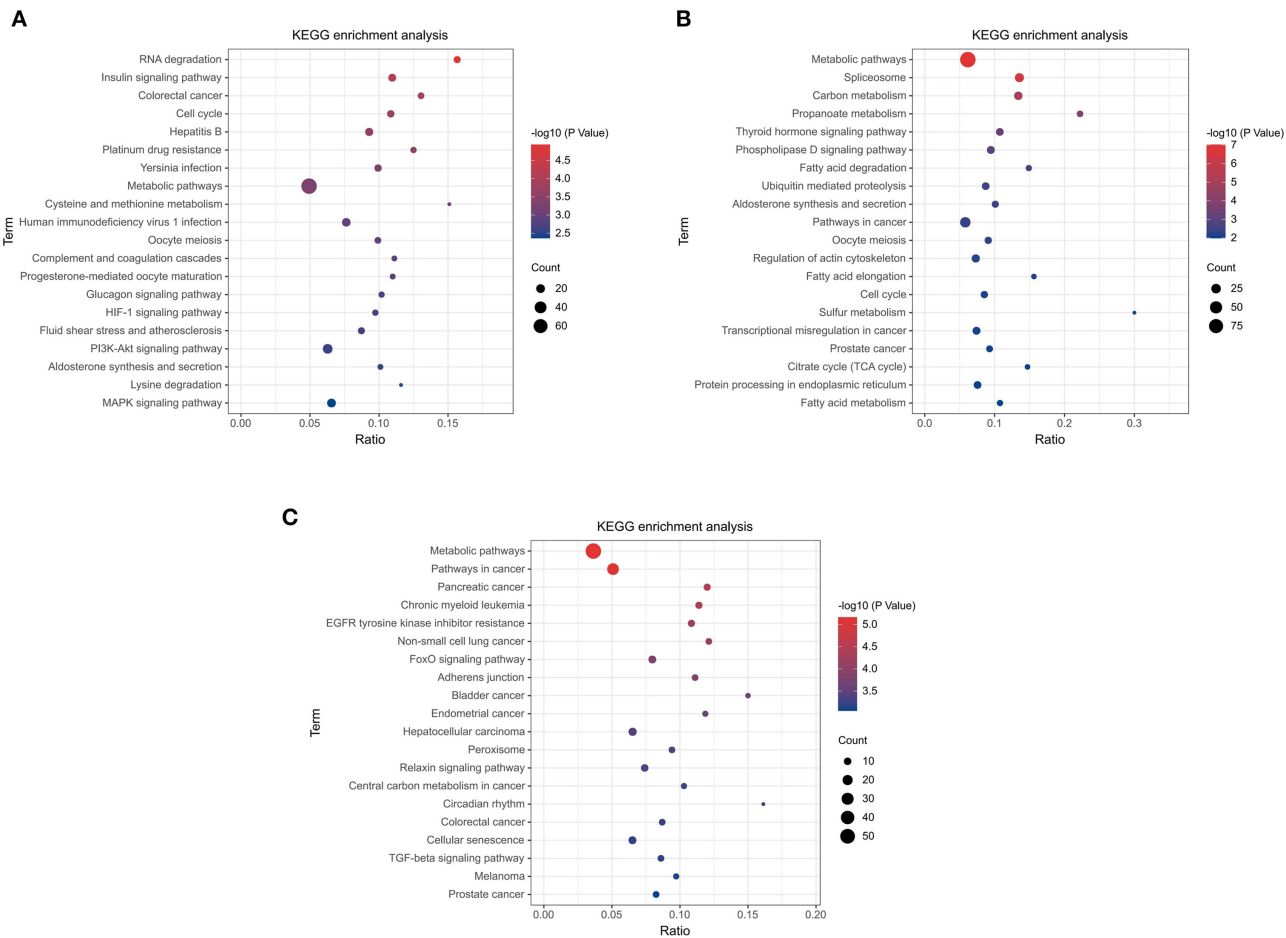
KEGG enrichment analysis of DElncRNAs and DEmRNAs. **(A)** QD-QP. **(B)** QE-QP. **(C)** QM-QP.

The top 20 pathways are shown in (Figures 5A–C). As a result, different KEGG signaling pathways associated with reproduction, cell proliferation, apoptosis and gonadotropin secretion were identified. In QD-QP, DEGs enrichment pathways include Cell cycle, Oocyte meiosis, PI3K-Akt signaling pathway and MAPK signaling pathway (Figure 5A). In the QE-QP group, DEGs were involved in Metabolic pathways, Oocyte meiosis, Cell cycle and other pathways (Figure 5B). In QM-QP, DEGs were significantly enriched in Metabolic pathways, Cellular senescence, and TGF-beta signaling pathway (Figure 5C). In the QD-QM, QE-QM, and QD-QE groups, these DEGs were significantly enriched in many pathways, including Metabolic pathways, Autophagy–animal, Apoptosis, MAPK signaling pathway, and TGF-beta signaling pathway (Supplementary Figure S4).

### Gene co-expression network construction

#### Clustering of mRNA co-expression modules

**Figure 6:**
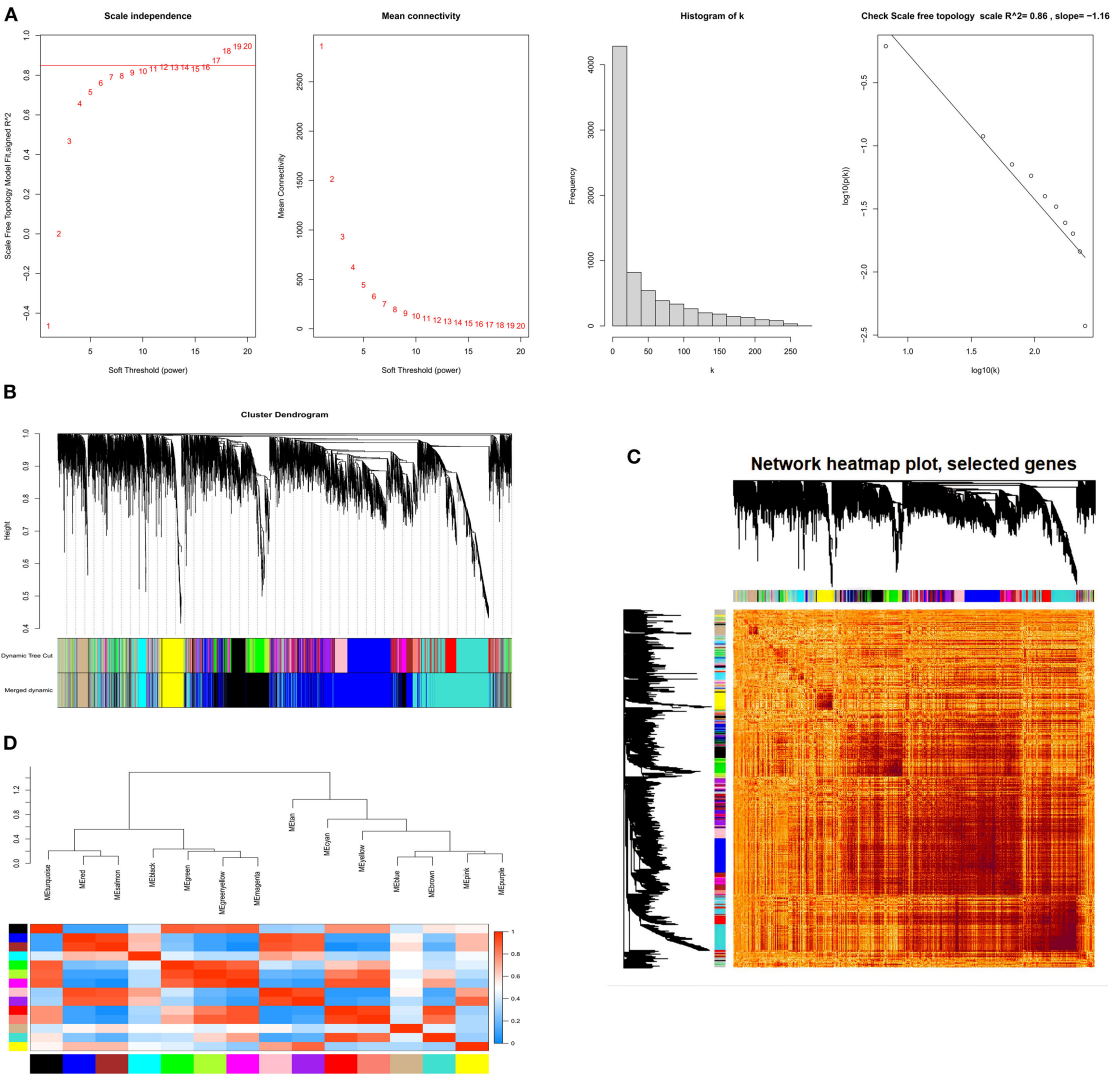
The process of screening follicular development mRNAs using WGCNA. **(A)** Network topology analysis for various soft-threshold powers. Scale-free topologies were examined; the adjacency matrix was defined using a soft threshold of β = 17. **(B)** Clustered dendrograms of genes, based on differences in topological overlap, and the specified module colors. **(C)** Heat map of the intergenic topological overlap matrix (TOM) based on co-expression modules. A redder background indicates a higher module correlation. **(D)** Visualization of gene networks using heat maps.

We further explored the genes involved in follicle development by constructing co-expression modules using the WGCNA software package tools. First, we filtered genes with minor or abnormal variants; then, based on R^2^ = 0.86, the best β = 17 was selected in the gene expression matrix to construct an approximately scale-free topological overlap matrix (Figure 6A). All selected genes were clustered using a topological overlap matrix (TOM)-based dissimilarity measure, which was based on a dynamic tree-cutting algorithm that divided the dynamic tree into seven modules each marked with different colors (Figure 6B). The number of genes in each module is shown in Supplementary Figure S5. Next, the Pearson correlation coefficient was used to analyze the interaction of these co-expression modules. Hierarchical clustering of eigengenes was performed on the modules in a cluster analysis, and branches (meta-modules) of the dendrogram were grouped based on the correlation of the eigengenes (Figure 6C). Each module contains a different gene cluster and is marked with a different color in the topologically overlapping heat map; red represents positive correlation and blue represents negative correlation (Figure 6D).

#### Clustering of lncRNA co-expression modules

**Figure 7:**
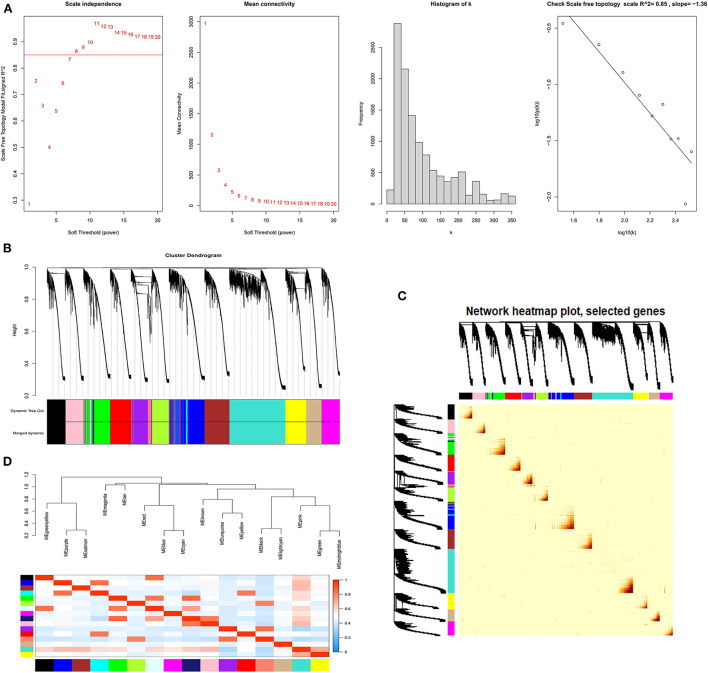
The process of screening follicular development LncRNAs using WGCNA. **(A)** Selecting for soft threshold (power). When the power value is 8, the degree of independence was > 0.85 for the first time. **(B)** A Clustering dendrogram of genes. Dissimilarity was based on the topological overlap, together with assigned module colors. The 17 coexpression modules are displayed in different colors. **(C)** Heat map of the intergenic topological overlap matrix (TOM) based on co-expression modules. A redder background indicates a higher module correlation. **(D)** Visualization of gene networks using heat maps.

In the lncRNA expression matrix, based on R^2^ = 0.86, a soft threshold of β = 8 was set to construct a scale-free network with a scale-free topological fit index > 0.85 (Figures 7A,B). We determined the final 17 modules based on average hierarchical clustering and dynamic tree cutting, and the number of genes in each module is shown in Supplementary Figure S6. As shown in Figure 7C, based on the correlations of the eigengenes, a heatmap of topological overlap was constructed while being marked with different colors (Figure 7D).

### Module–trait relationship analysis

**Figure 8:**
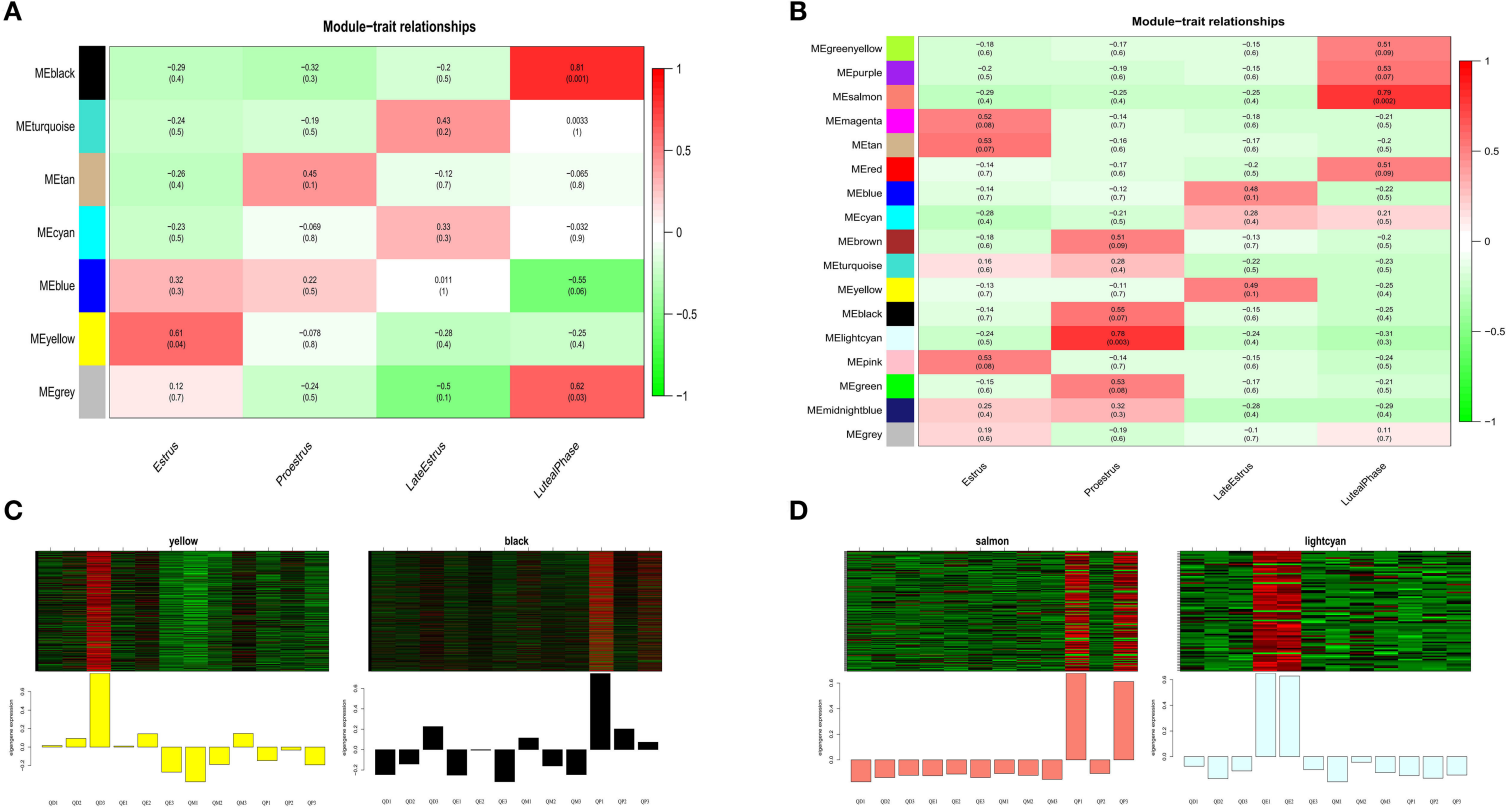
Key module analysis. mRNA module-feature relationships **(A)**, and LncRNA module-feature relationships **(B)**, The number of each grid indicates the module-feature correlation, and the number in parentheses indicates the p-value. **(C)** Heat map gene expression patterns of mRNA key modules. **(D)** LncRNA key modules. The upper panel shows the heat map of gene expression in modules in different samples, and the lower panel shows the expression pattern of module feature values in different samples.

We summarized the gene co-expression of eigengenes and calculated the correlation of each eigengene with each stage of the estrous cycle, i.e., estrus, late estrus, luteal phase, and proestrus, which was determined by ME, the principal components of gene expression in the module, and the Spearman correlation coefficients the stages of the estrous cycle. Module-trait relationship analysis showed that in the mRNA co-expression relationship graph, genes in the black module (cor = 0.81, *P*<0.001) were positively correlated with the luteal phase, and genes in the yellow module (cor = 0.61, *P*<0.04) were positively correlated with estrus (Figure 8A). In the lncRNA co-expression relationship map, the genes in the lightcyan module (cor = 0.79, *P*<0.002) and the genes in the salmon module (cor = 0.78, *P* < 0.003) were significantly positively correlated with the pre-estrus and luteal phases, respectively (Figure 8B). Genes in these modules may be associated with follicular development.

Next, we analyzed the gene expression patterns of the four modules in detail and identified specific expression patterns at different stages. Gene expression of the black and salmon modules was elevated generally during the luteal phase. During the estrus and pre-estrus stages, the gene expression of the yellow and lightcyan modules was typically higher (Figures 8C,D).

### Functional enrichment analysis of key modules

**Figure 9:**
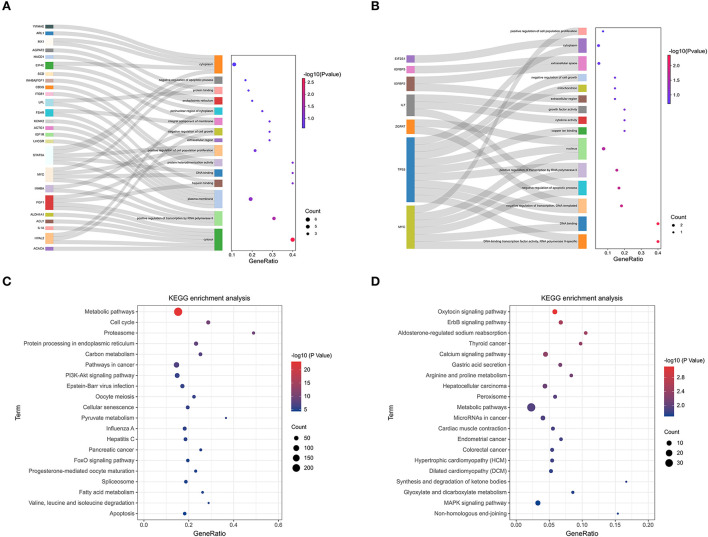
KEGG pathway enrichment analysis of key module. **(A)** GO analysis of the black module and yellow module. **(B)** GO analysis of the light cyan module and salmon module. **(C)** KEGG enrichment analysis of black module and yellow module. **(D)** KEGG enrichment analysis of the lightcyan module and salmon module.

Follicular developmental processes and pathways can be shared and differentially regulated in sheep. GO enrichment analysis showed that genes in the black and yellow modules were enriched in the positive regulation of transcription by RNA polymerase II, positive regulation of cell population proliferation, negative regulation of cell growth, negative regulation of apoptotic process, positive regulation of transcription, DNA-templated and DNA-binding transcription factor activity, RNA polymerase II-specific (Figure 9A). Genes in the lightcyan and salmon modules were enriched in the negative regulation of transcription, negative regulation of apoptotic process, positive regulation of transcription by RNA polymerase II, cytokine activity and growth factor activity (Figure 9B). In addition, different KEGG signaling pathways related to reproduction, cell proliferation, cell cycle and gonadal hormone secretion were identified. In black and yellow modules, genes were significantly enriched in Metabolic pathways, Cell cycle, PI3K-Akt signaling pathway, Oocyte meiosis, Cellular senescence, Progesterone-mediated oocyte maturation and Apoptosis pathways (Figure 9C). In KEGG signaling pathway analysis of lncRNA target genes of lightcyan and salmon modules, associations with reproduction and cell proliferation, differentiation, and migration were identified. These include: Calcium signaling pathway, Metabolic pathways, MAPK signaling pathway, and PI3K-Akt signaling pathway (Figure 9D).

### Identification and analysis of hub genes

#### PPI network analysis and hub gene identification

**Figure 10:**
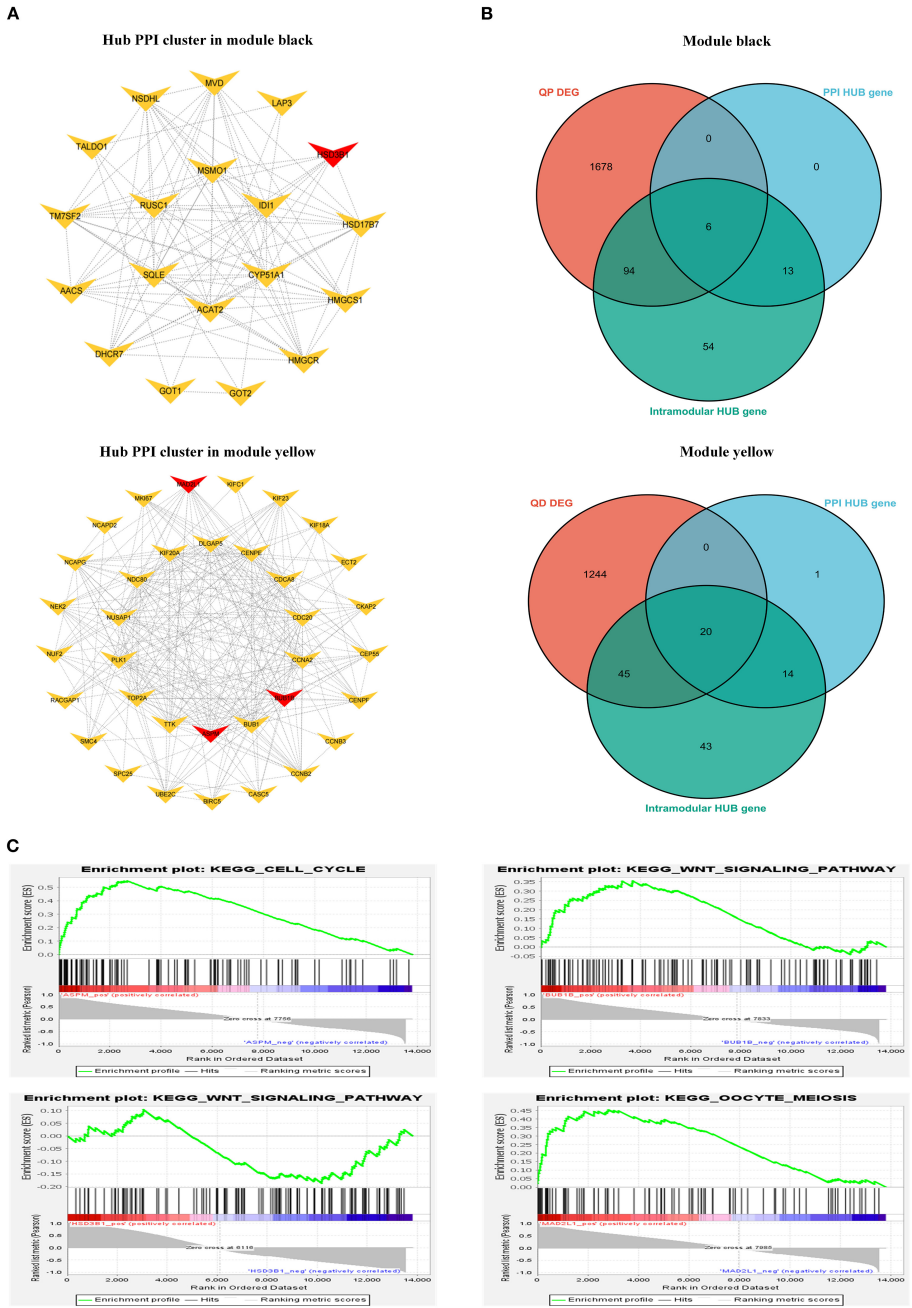
Identification of hub genes. **(A)** Typical hub cluster of the PPI network in module black and yellow. **(B)** Wayne diagram of overlapped genes between PPI hub cluster genes, DEmRNAs and intra-module hub genes. **(C)** GSEA enrichment plots in *BUB1B, MAD2L1, ASPM*, and *HSD3B1*.

We constructed a PPI network from the STRING database to explore gene interactions in the modules (Supplementary Figure S7). Hub gene clusters scoring higher than 3 in each PPI network were identified using the Cytoscape MCODE plug-in (Figure 10A). The “Ballgown” package was used to study DEGs between genes and other stages at each estrus stage time point, with thresholds of *P* < 0.05 and |log (FC)|> 1. The intra-module hub genes in each module are listed in Supplementary Table S5. PPI hub cluster genes, DEGs, and highly connected overlapping genes were in their respective modules at each stage time point. Based on our results, we focused on the following four hub genes: the *BUB1B, MAD2L1*, and *ASPM* genes in the yellow module and the *HSD3B1* gene in the black module (Figure 10B). In addition, GSEA was performed to explore the potential regulatory mechanisms of *BUB1B, MAD2L1, ASPM*, and *HSD3B1*. The results showed that these genes were functionally enriched in cell cycle, oocyte meiosis, and WNT signaling pathways (Figure 10C).

#### Identification of central lncRNA

**Figure 11:**
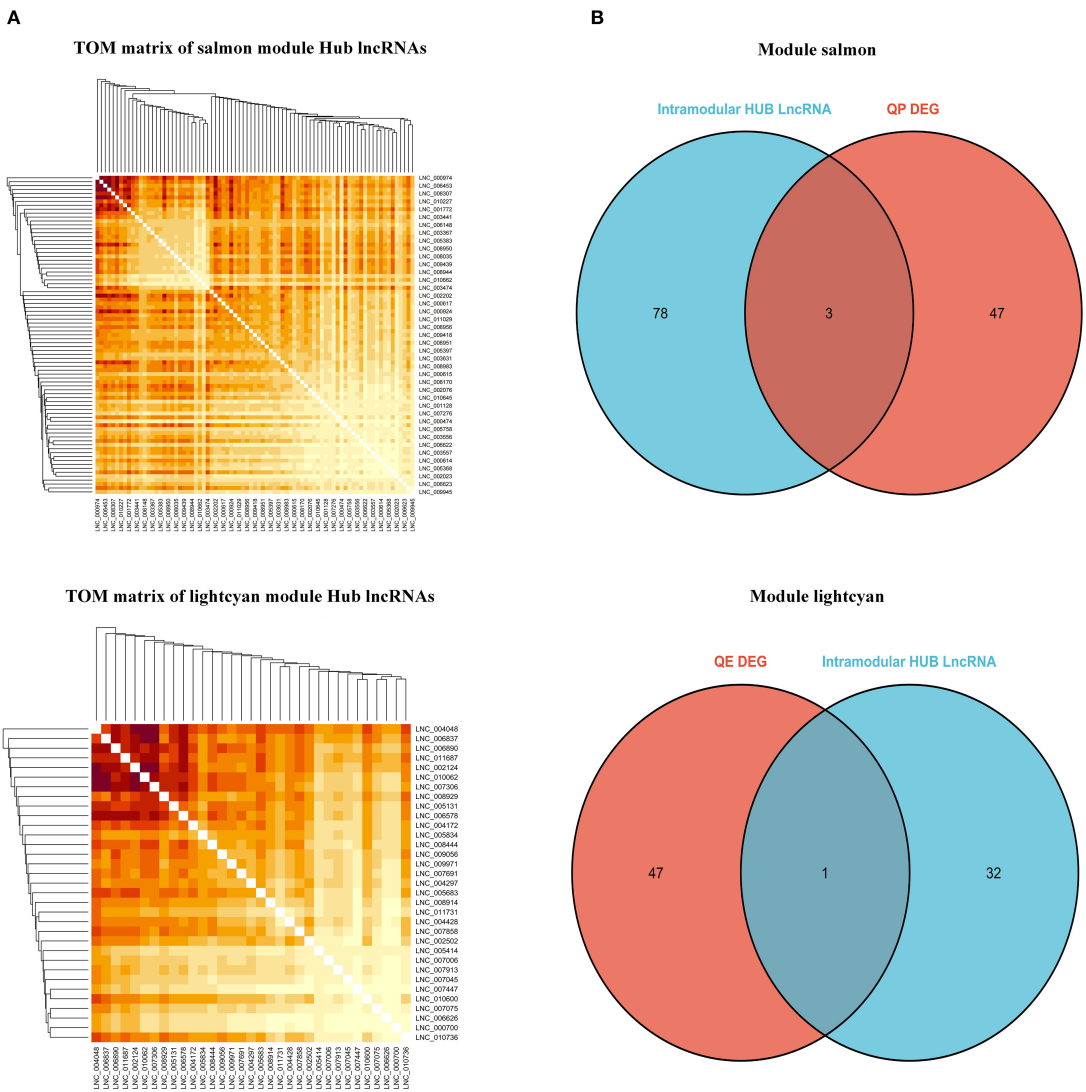
Identification of hub LncRNAs. **(A)** TOM matrix between candidate LncRNAs. **(B)** Wayne diagram of overlapped genes between LncRNAs and intra-module hub genes.

By filtering under the thresholds of MM>0.8 and GS>0.8, the intra-module hub genes in each module are listed in Supplementary Table S6. The TOM matrix among candidate lncRNAs was drawn based on the significance and degree of difference in fold change (Figure 11A). DElncRNAs in each stage overlapped with highly connected LncRNAs in the respective modules. Our results show that *LNC_006453, LNC_005683, LNC_003443*, and *LNC_003367* overlap (Figure 11B).

### TF forecasting and analysis in related modules

**Figure 12:**
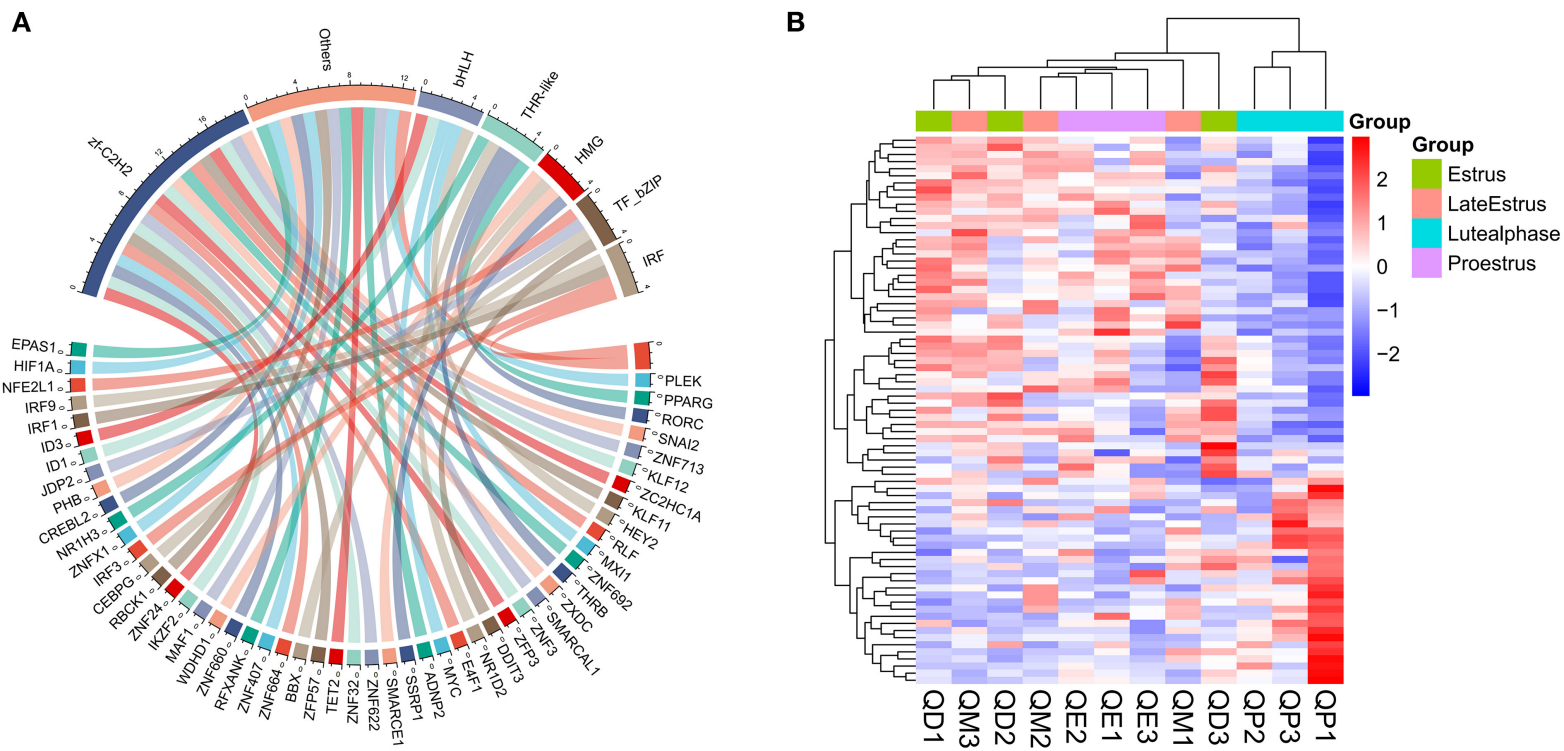
TF family statistics and heatmap analysis in black and yellow modules. **(A)** TF prediction and family statistics of three modules. **(B)** Heatmap of TFs in three modules. Red and Blue represent up- and down-regulated DEmRNAs, respectively.

We aligned the putative protein sequences with the animal TFdb database for TF prediction. A total of 1302 expressed TFs belonging to 71 TF families were identified (Supplementary Figure S8). TFs play an important role in follicular development. For example, the transcription factor *RUNX1* improves estrogen secretion by regulating the expression of genes related to steroidogenesis (FSHR, LHR, etc.), stimulates cell proliferation in ovarian granulosa cells, and promotes follicle growth and maturation (29). Our WGCNA results indicated that the expression of central genes in the above-mentioned black and yellow modules was associated with follicular development. We identified 79 TFs belonging to 25 TF families in these two modules. The most abundant TF families were zf-C2H2, bHLH, THR-like, HMG, IRF, and TF_bZIP (Figures 12A,B).

**Figure 13:**
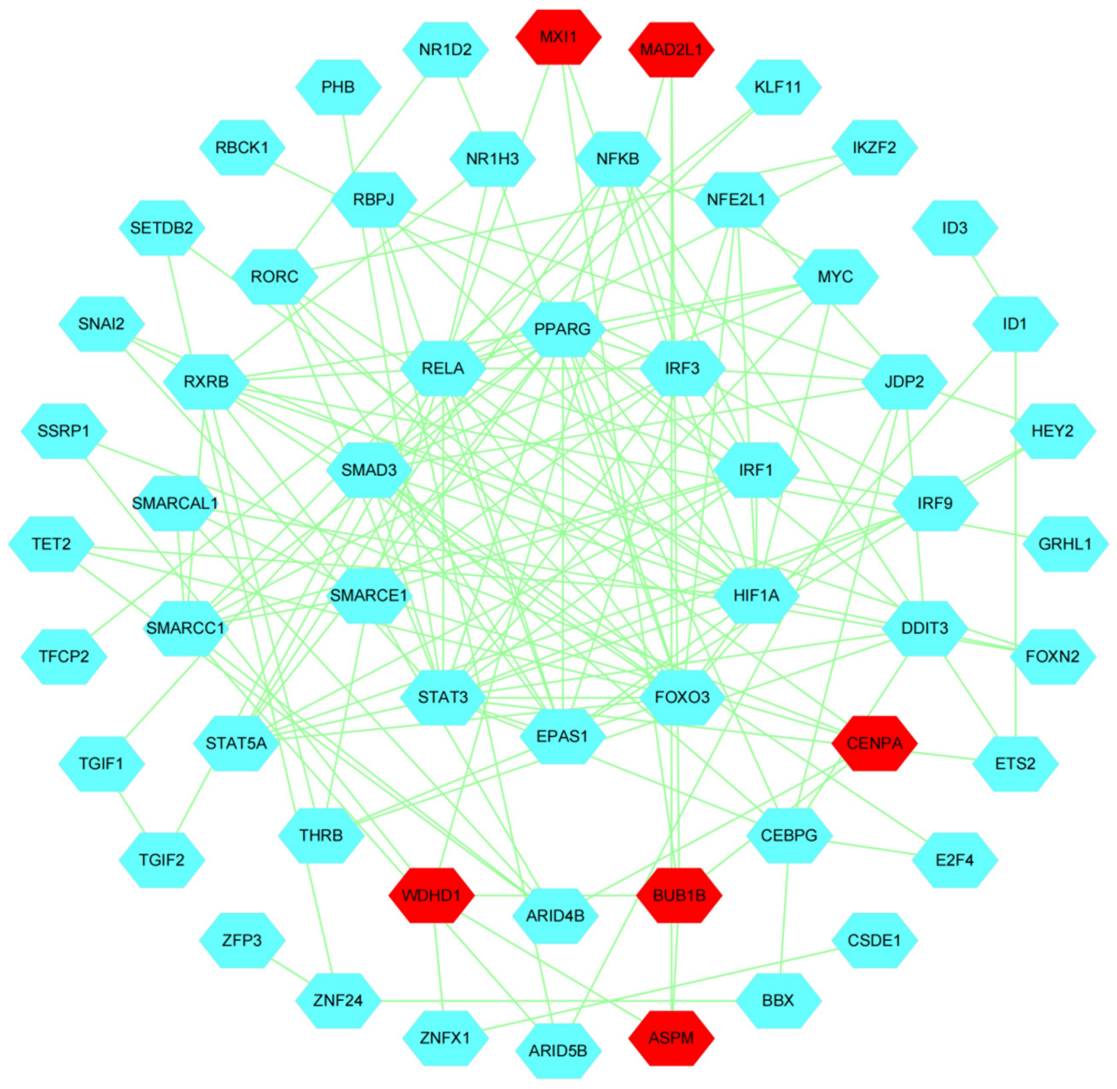
Construction of Hub genes and TFs regulation networks about follicular development pathway by Cytoscape software. The Red and cyan hexagons represent identified hub genes involved in the network.

Next, we performed network analysis to investigate the interactions between hub genes and TFs involved in follicle development. A regulatory network of HUB genes and TFs was constructed using Cytoscape software, with 57 nodes, 150 edges, and six hub genes (*BUB1B, MAD2L1, ASPM, WDHD1, CENPA, MXI1*). Our network analysis indicated (Figure 13) that TFs in modules may be key regulators during follicle development.GSEA analysis indicated that module *WDHD1, CENPA*, and *MXI1* genes may regulate follicle and maturation through the cell cycle and GnRH signaling pathway, P53 signaling pathway, etc., but this finding needs further validation (Supplementary Figure S9).

## Discussion

WGCNA is a systems biology method used to describe gene association patterns between different samples. Analyzing the associations between genes and dividing them into multiple modules based on their expression patterns, and then analyzing them in modules, reduces the computational workload and increases accuracy. The difference between WGCNA and Differential Gene Analysis (DEG) is that DEG mainly analyzes sample-to-sample differences, whereas WGCNA considers not only individual gene functions but also the relationships between genes.

We used WGCNA to probe gene association patterns and assess potential interactions between expressed genes. Two modules (the black module in the luteal phase and the yellow module in the estrus phase) were determined to be highly correlated with the estrus cycle. KEGG analysis of both module genes found significant enrichment in the cell cycle, PI3K-Akt signaling pathway, and Oocyte meiosis. During reproduction, regulating cell cycle-related factors contributes to better follicle development and protects female fertility (30, 31). Activation of the PI3K-Akt signaling pathway and Oocyte meiosis can promote oocyte maturation (32–34). Our results suggest that the cell cycle, PI3K-Akt signaling pathway, and Oocyte meiosis play important roles in follicle development.

In our study, WGCNA results identified 7 hub genes (*BUB1B, MAD2L1, ASPM, WDHD1, CENPA, MXI1*, and *HSD3B1*). Steroid hormones are essential for follicular development, and HSD3B1 is a steroid hormone metabolism gene involved in progesterone biosynthesis (35). *HSD3B1* is known to be progressively upregulated after ovulation and peaks during the luteal phase (36, 37), which is consistent with the findings of this study. Our results identified the *HSD3B1* gene affecting follicle development as a central gene, which further demonstrated the high reliability of our transcriptome analysis. *MAD2L1* accumulates early in oocyte maturation (38) and affects cell proliferation and cell cycle progression (39). Reduced levels of *MAD2L1* expression have been shown to lead to a shortened duration of meiotic I and meiotic spindle abnormalities, promoting oocyte maturation (40). In this study, *MAD2L1* expression was downregulated from the luteal phase to estrus, indicating that *MAD2L1* plays an important role in oocyte maturation during the estrous cycle in sheep. *BUB1B* is essential for mammalian meiosis and is an important factor necessary for follicular development (41). Complete loss of BUB1B reduces ovarian function and fertility in female mice (42). *ASPM* is a spindle pole intermediate protein that regulates reproduction in female mammals (43). Loss of *ASPM* results in abnormal ovarian function while preventing folliculogenesis (44). Silencing ASPM causes cell cycle arrest and leads to apoptosis (43). This study found that *ASPM* was significantly higher in the estrous phase than in the luteal and pre-estrous phases, suggesting that *ASPM* may have an integral role in follicular development by regulating cell proliferation and the cell cycle.

The transcription factor *WDHD1* is involved in chromatin assembly, transcription, and replication (45). To clarify its mechanism, we performed GSEA analysis, which showed that it was mainly enriched in oocyte meiosis and cell cycle, which is consistent with current studies on the WDHD1 mechanism. In addition, *WDHD1* has been reported to affect cell proliferation, apoptosis, and cell cycle through transcriptional regulation of target gene Skp2 expression (46). *CENPA*, also known as the histone H3 variant (*CenH3*), is localized as a marker of centromeric components (47). As previously reported, the transcription factor *CENPA* mediates an important role for *MYBL2* in ovarian cancer cell proliferation (48). *MAX* interactor 1 (*Mxi1*), a member of the mitotic arrest defect (*MAD*) family, can be regulated at the transcriptional level (49). Mxi1 has been reported to promote cell proliferation through the IL-8 and ERK1/2 pathways (50), but no studies have reported the function of this gene in the ovary. Therefore, this study speculates that *WDHD1, CENPA*, and *Mxi1* act as potential regulators of other genes, alter the expression pattern of reproduction-related genes, and have a positive effect on follicle development in sheep, thereby affecting the multiparity of Cele black sheep, but this finding needs further validation.

## Conclusion

In this study, we used WGCNA analysis to identify important genes regulating follicle development in multiparous sheep. Our results indicate that “Cell cycle”, “PI3K-Akt signaling pathway”, and “Oocyte meiosis pathway” play key roles in multiparous reproduction. We also identified seven genes that may be central to this mechanism. Overall, our RNA-seq data provide an alternative strategy and a valuable resource for investigations.

## Data availability statement

The data presented in the study are deposited in the SRA repository, accession number-PRJNA905555.

## Ethics statement

The animal study was reviewed and approved by the animal study was reviewed and approved by the Research Committee of the First Hospital of Shihezi University (A2016-085).

## Author contributions

XZ conducted the experiments. JW and XZ designed the study, analyzed the data, and drafted the manuscript. HC conducted parts of the experiments and collected samples. All authors read and approved the final manuscript.
