# Supplementary material for: Identification of hub genes associated with follicle development in multiple births sheep by WGCNA
Source: Front Vet Sci. 2022 Dec 19;9:1057282. doi: 10.3389/fvets.2022.1057282 (PMC9806849; doi:10.3389/fvets.2022.1057282)
Supplement: Supplementary file 7 [file Data_Sheet_1.pdf]

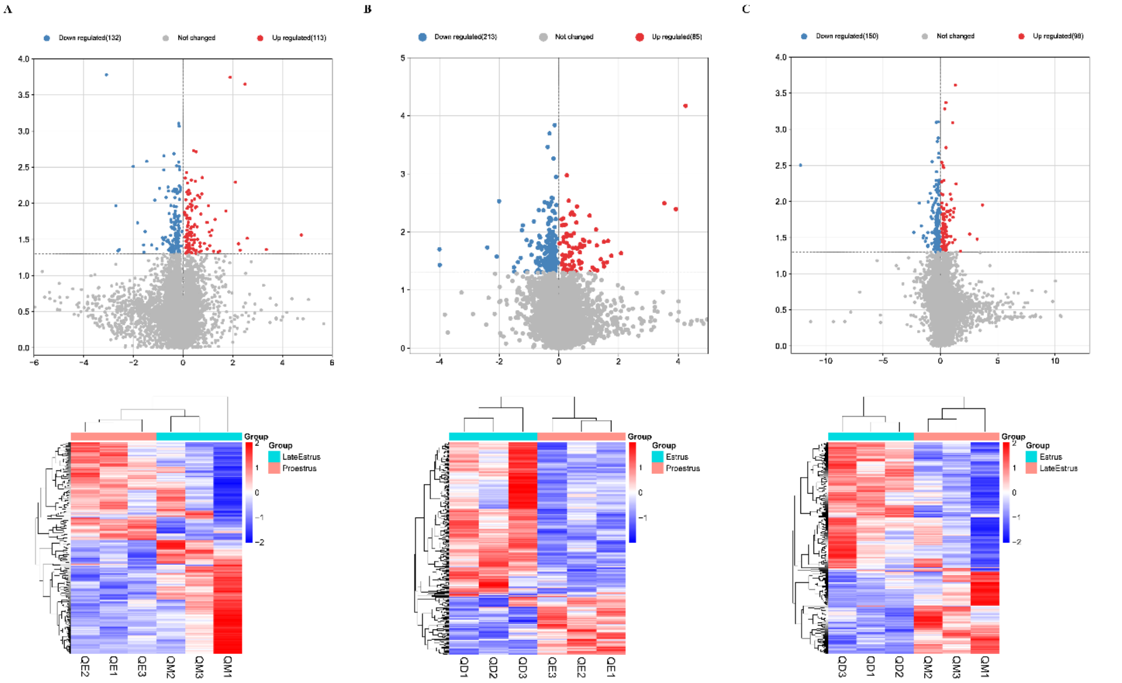

Supplementary Figure 1. Graphical representation of DEmRNAs at each stage of the estrous cycle in sheep.The Volcano map and Hierarchical clustering analysis of DEmRNAs in the QE-QM (A), QD-QE(B), and QD-QM (C).

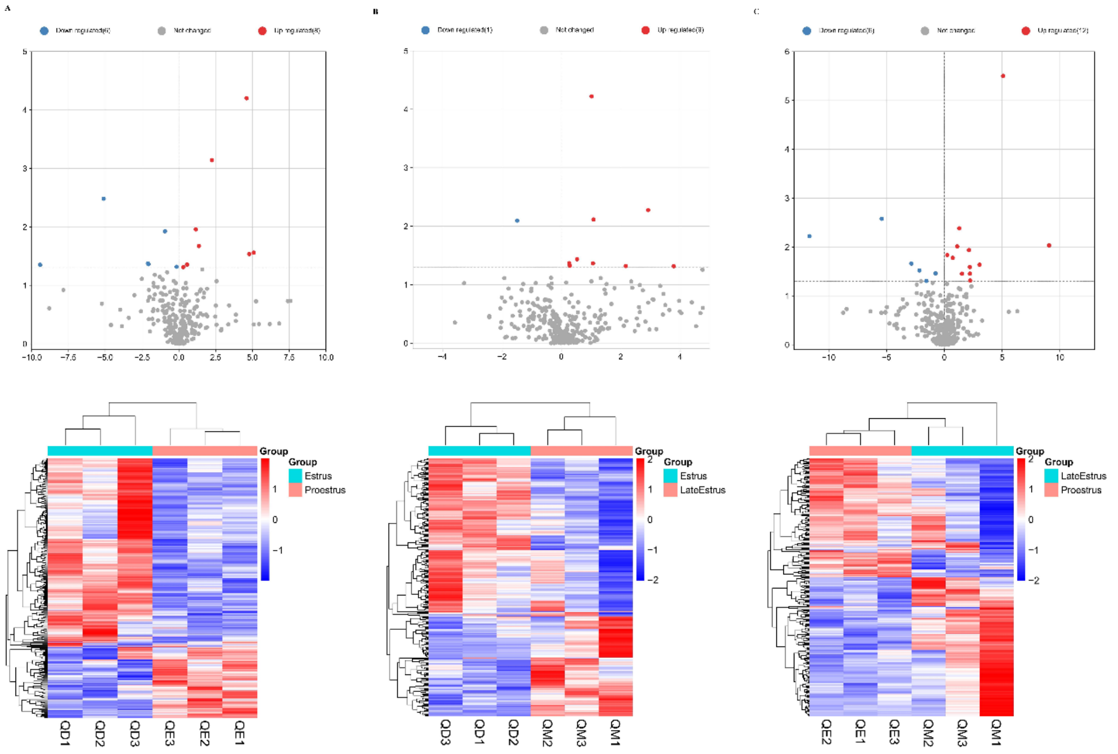

Supplementary Figure 2. Graphical representation of DELncRNAs at each stage of the estrous cycle in sheep. The Volcano map and Hierarchical clustering analysis of DELncRNAs in the QE-QM (A), QD-QE(B), and QD-QM (C).

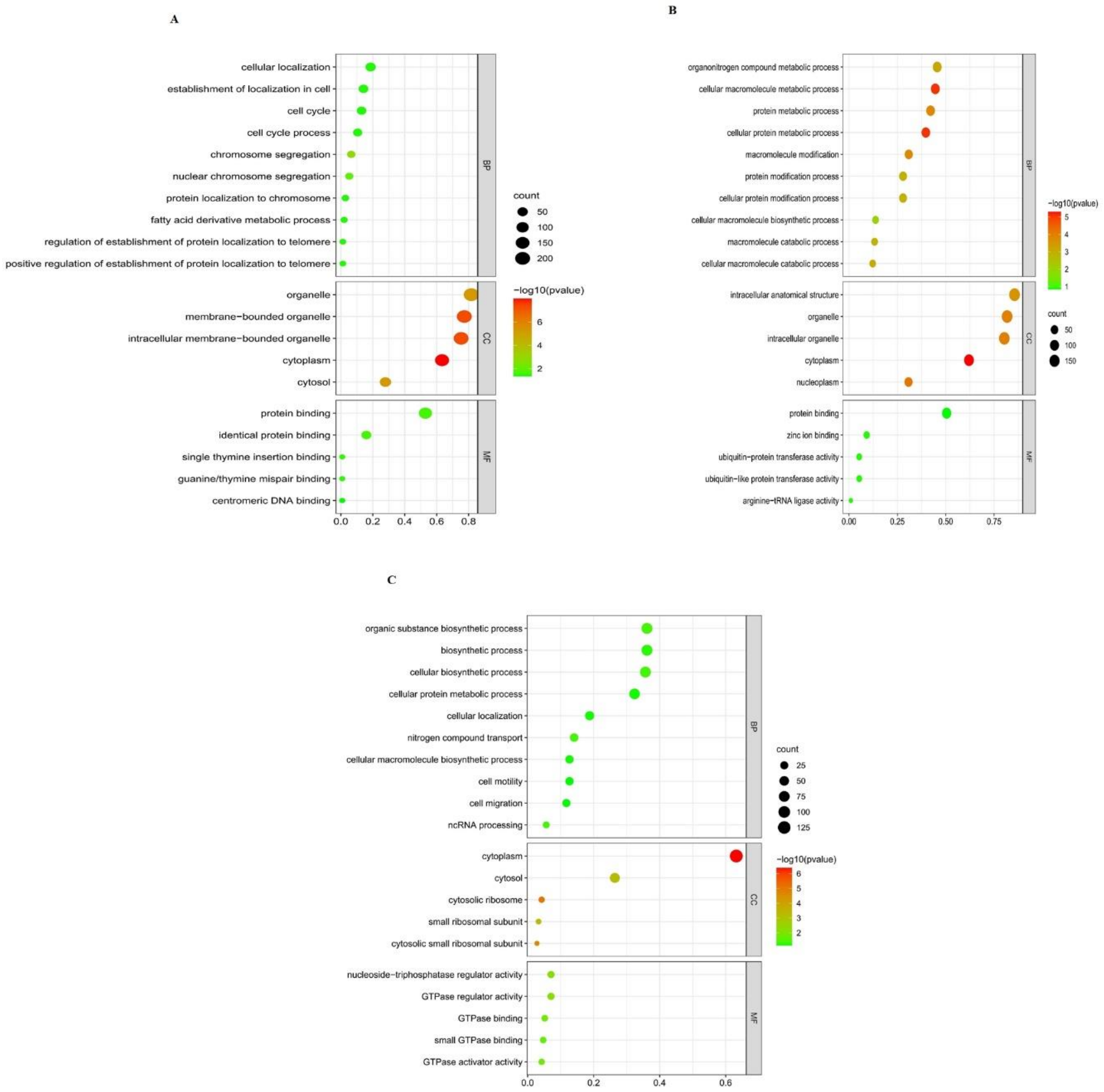

Supplementary Figure 3. GO enrichment analysis of DElncRNAs and DEmRNAs. (A) QD-QE; (B) QD-QM; (C) QE-QM.

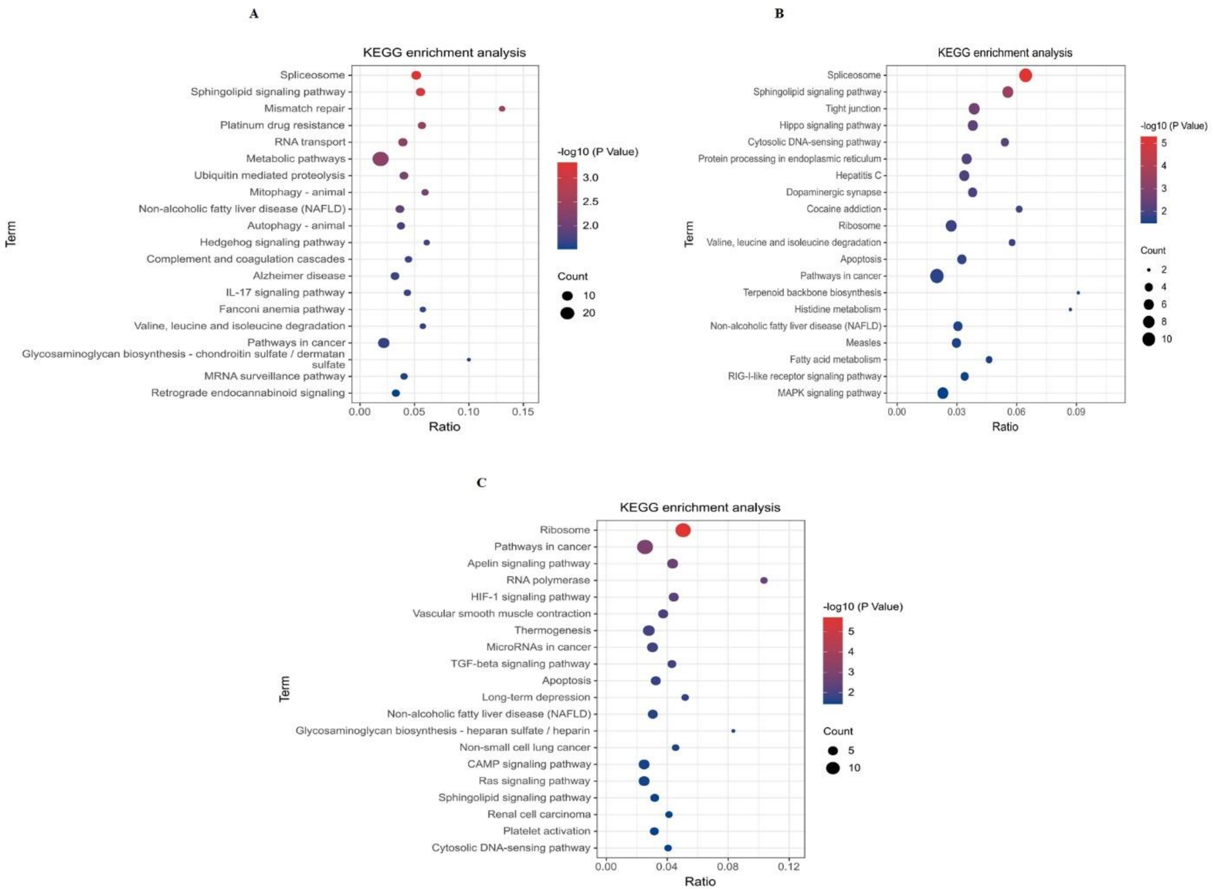

Supplementary Figure 4. KEGG enrichment analysis of DElncRNAs and DEmRNAs. (A) QD-QE; (B) QD-QM; (C) QE-QM.

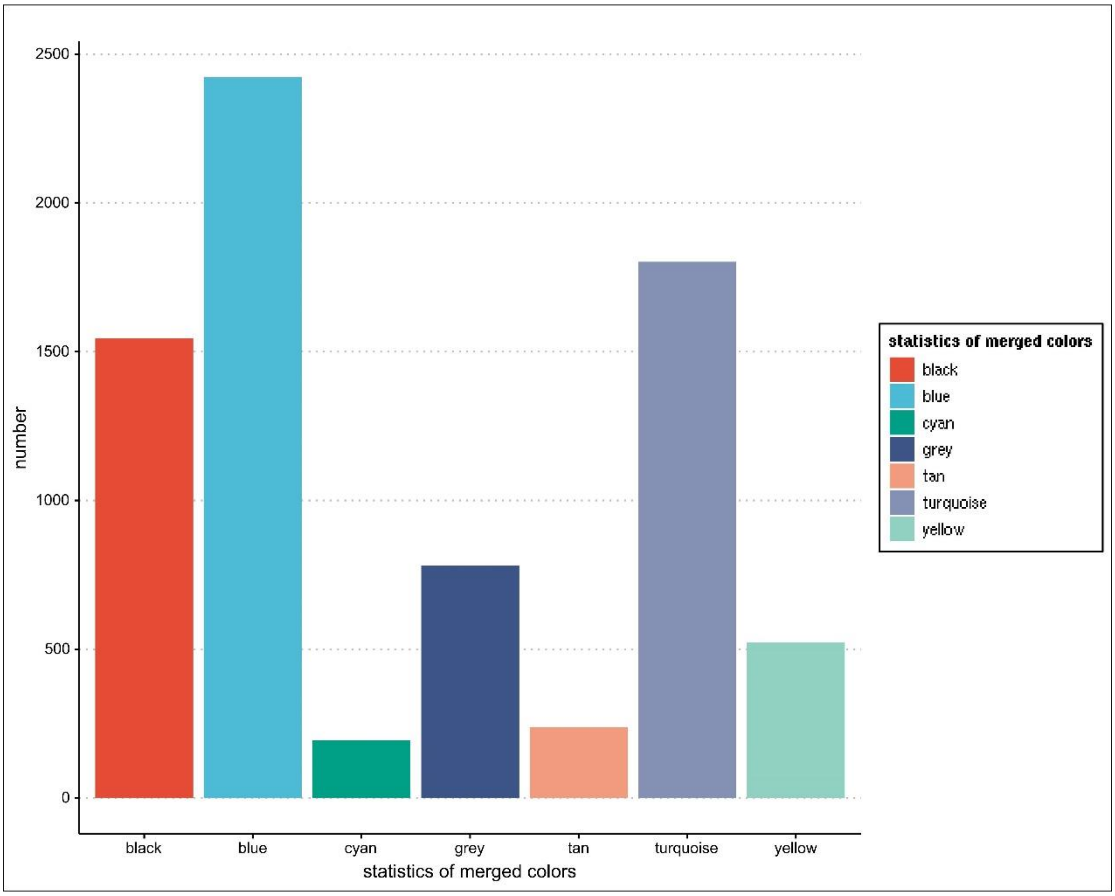

Supplementary Figure 5. The number of mRNAs in the co-expression modules.

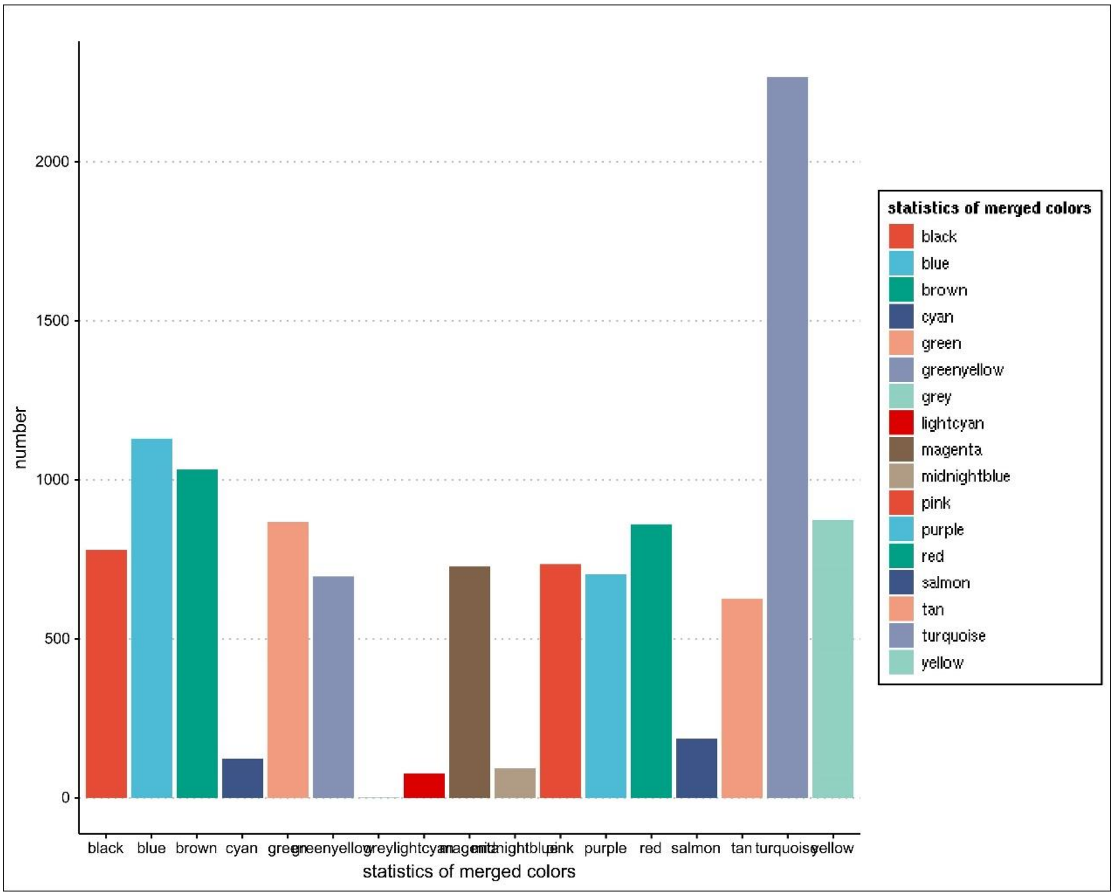

Supplementary Figure 6. The number of LncRNAs in the co-expression modules.

A B

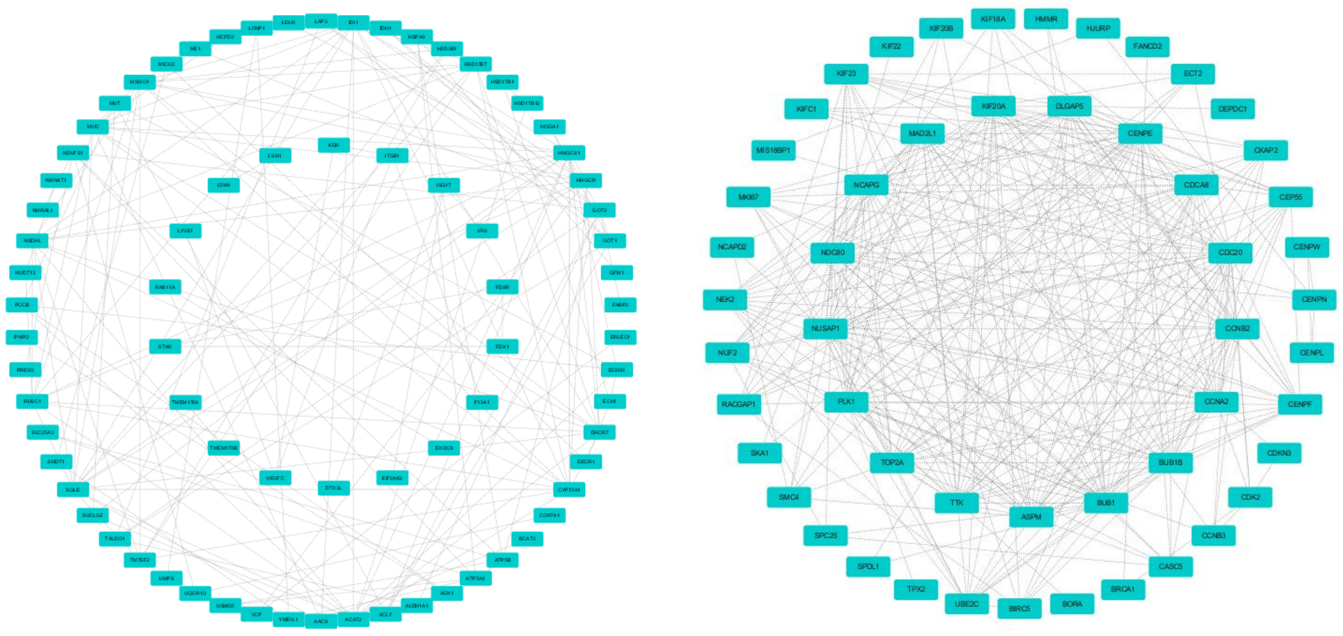

Supplementary Figure 7. (A) All black modules PPI analysis; (B) All yellow modules PPI analysis.

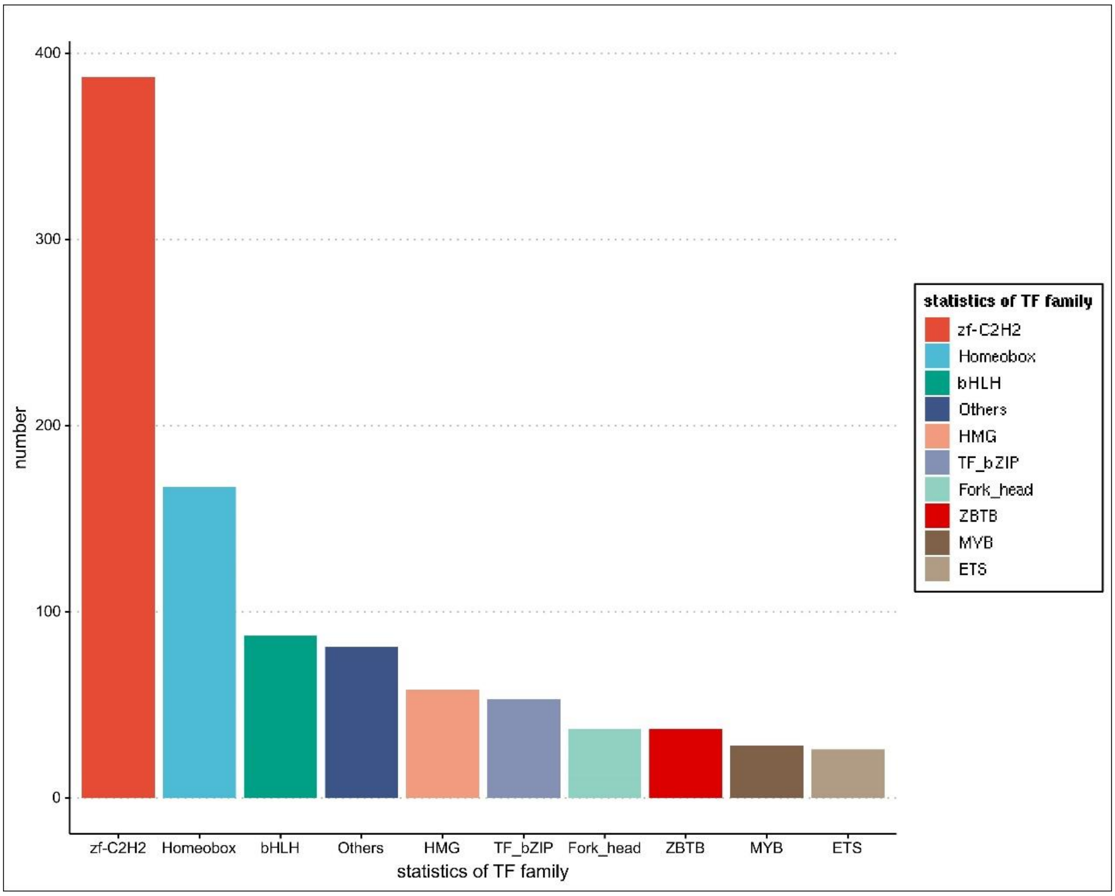

Supplementary Figure 8.TF prediction and statistics of TF family.

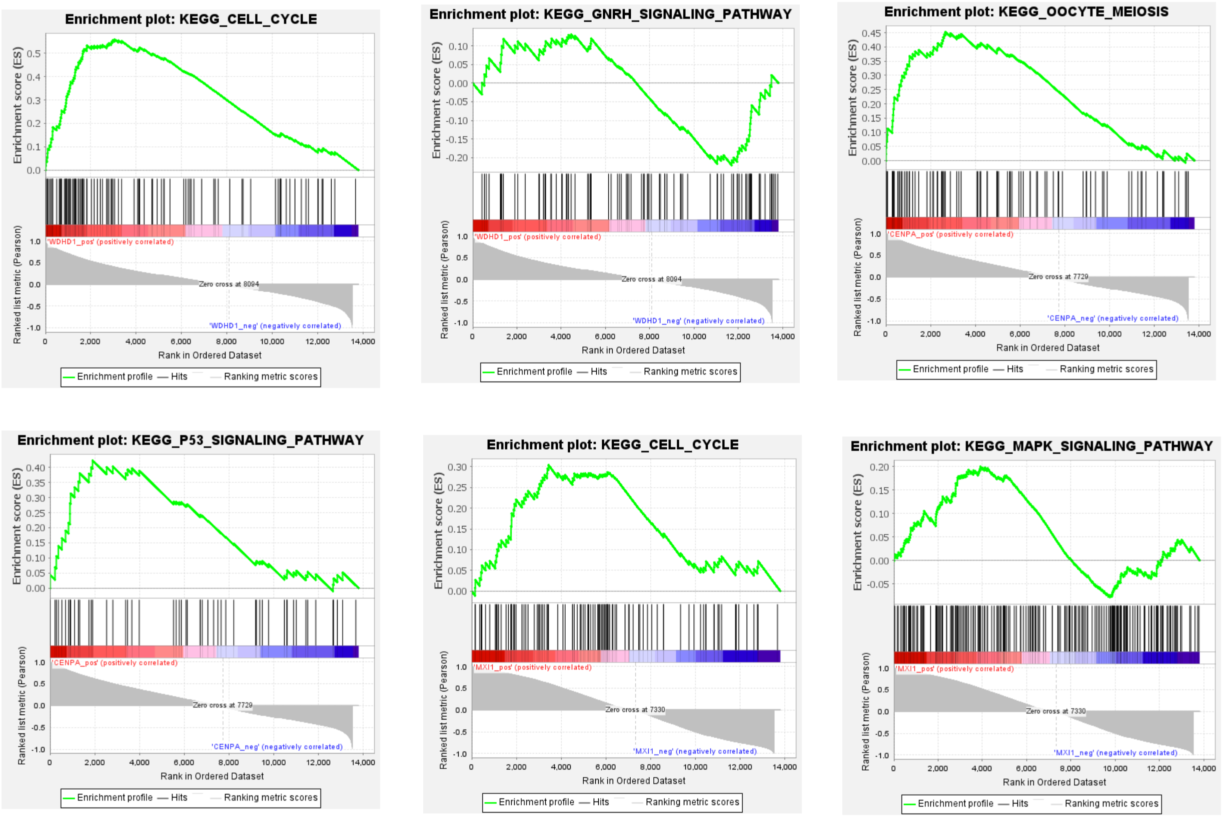

Supplementary Figure 9. GSEA analysis of key genes *WDHD1, CENPA, MXI1*.
